# Upregulation of lncRNA ZFAS1 promotes lung adenocarcinoma progression by sponging miR‐1271‐5p and upregulating FRS2

**DOI:** 10.1111/1759-7714.13525

**Published:** 2020-06-08

**Authors:** Gang Fan, Jin Jiao, Feng Shen, Fulu Chu

**Affiliations:** ^1^ Department of Clinical Laboratory Shandong Provincial Hospital Affiliated to Shandong First Medical University Jinan China; ^2^ Department of Clinical Laboratory Shandong Maternal and Child Health Care Hospital Jinan China

**Keywords:** FRS2, lncRNA ZFAS1, lung adenocarcinoma, miR‐1271‐5p

## Abstract

**Background:**

Nowadays, the important roles of long non‐coding RNAs (LncRNAs) in lung adenocarcinoma (LAD) is being increasingly recognized. The purpose of this study was to explore the regulatory mechanism of lncRNA ZFAS1 in LAD.

**Methods:**

The expression and function of lncRNA ZFAS1 were assessed by RT‐qPCR, CCK‐8, transwell and dual luciferase reporter assays.

**Results:**

Upregulation of lncRNA ZFAS1 was found in LAD tissues and cells. Knockdown of lncRNA ZFAS1 restrained cell proliferation, migration and invasion in LAD cells. In addition, we determined that lncRNA ZFAS1 could directly bind to miR‐1271‐5p. MiR‐1271‐5p functioned as a tumor suppressor in LAD, and lncRNA ZFAS1 promoted LAD development by downregulating miR‐1271‐5p. Furthermore, FRS2 was a direct target of miR‐1271‐5p. FRS2 promoted progression of LAD by mediating lncRNA ZFAS1/miR‐1271‐5p axis.

**Conclusions:**

LncRNA ZFAS1 promotes cell proliferation, migration and invasion in LAD by downregulating miR‐1271‐5p or upregulating FRS2.

## Introduction

Lung cancer is the malignant tumor that is most harmful to human health and life in the world today. The results of epidemiological investigations in recent years show that the incidence of lung adenocarcinoma (LAD) in many countries (including China), whether in smokers or non‐smokers, has surpassed lung squamous cell carcinoma (LSCC).^1^ Compared with LSCC, LAD is prone to distant hematogenous metastasis and hematogenous lymphatic metastasis, and the prognosis of LAD patients is relatively poor.^2^ In the past, the median survival time for patients with advanced LAD is less than one year. With the improvement in treatment methods, the median survival of advanced patients treated with targeted therapy and immunotherapy now reaches more than four years.^3^ However, few effective therapeutic targets are available. Therefore, it is still important to explore new targets for diagnosis and treatment of patients with LAD.

Long non‐coding RNAs (lncRNAs) are a class of RNAs defined as being transcripts with lengths exceeding 200 nucleotides and do not show any protein‐coding potential, accounting for a significant proportion of total non‐coding RNAs (ncRNA).^4^ It has been reported that lncRNAs are spatially correlated with transcription factors and regulate lung development.^5^ Meanwhile, the specific roles of lncRNAs are also found in LAD. For example, lncRNA XIST was shown to expedite LAD progression through upregulating MDM2 expression via binding to miR‐363‐3p,^6^ and silencing of LINC00461 enhanced radiosensitivity of LAD cells by downregulating HOXA10 via miR‐195.^7^ LncRNA ZFAS1 has been demonstrated to participate in human cancers. Upregulation of lncRNA ZFAS1 has been identified in nasopharyngeal carcinoma and osteosarcoma.^8^, ^9^ Functionally, lncRNA ZFAS1 promotes esophageal squamous cell carcinoma (ESCC) cell proliferation, invasion, migration and induced apoptosis.^10^ In particular, lncRNA ZFAS1 has been reported to act as an oncogene by targeting miR‐193a‐3p in human non‐small cell lung cancer (NSCLC).^11^ However, the specific role of lncRNA ZFAS1 in LAD has not been completely confirmed. Therefore, this study was designed to solve the above problem.

In addition, miR‐1271‐5p has been predicted to have a binding site with lncRNA ZFAS1. MiR‐1271 has also been found to inhibit cell proliferation and metastasis by targeting LDHA in endometrial cancer,^12^ and miR‐1271 functioned as a metastasis and epithelial‐mesenchymal transition (EMT) inhibitor in human hepatocellular carcinoma.^13^ However, the role of miR‐1271‐5p remains largely unknown in LAD. Further, fibroblast growth factor receptor substrate 2 (FRS2) was found to be a potential target of miR‐1271‐5p. High expression of FRS2 has been identified in nephroblastoma and breast cancer.^14^, ^15^ It has also been reported that FRS2 is oncogenic in high‐grade serous ovarian cancer.^16^ However, the function of FRS2 has not been investigated in LAD.

In this study, the abnormal expressions and roles of lncRNA ZFAS1, miR‐1271‐5p, and FRS2 was measured in LAD. The relationship between lncRNA ZFAS1 and miR‐1271‐5p or FRS2 was also confirmed in LAD. The regulatory mechanism of lncRNA ZFAS1 in LAD was preliminarily illuminated in our research.

## Methods

### Clinical specimens

A total of 46 LAD tissues and adjacent healthy lung tissues were collected from Shandong Provincial Hospital affiliated to Shandong First Medical University. The LAD patients who participated in this study provided their written informed consents. All patients had no history of cancer and had not received chemotherapy or radiation therapy before surgery. Tissues were frozen in liquid nitrogen and stored in a −80°C refrigerator. The experiment was approved by the Institutional Ethics Committee of Shandong Provincial Hospital affiliated to Shandong First Medical University. The clinicopathological characteristics of the LAD patients are summarized in Table 1.

**Table 1:** The relationship between lncRNA ZFAS1 and the clinicopathologic characteristics in LAD

Characteristics	Number of cases (*n* = 46)	ZFAS1		*P*‐value
		High (*n* = 32)	Low (*n* = 14)	
Age (years)				0.45
≥ 60	19	14	5	
<60	27	18	9	
Gender				0.61
Male	28	20	8	
Female	18	12	6	
Tumor size (cm)				0.36
≥ 3	32	23	9	
<3	14	9	5	
Lymph node metastasis				0.03^*^
Absent	30	22	8	
Present	16	10	6	
TNM stage				0.01^*^
I + II	13	8	5	
III + IV	33	24	9	
Differentiation				0.07
Well	14	9	5	
Moderate‐poor	32	23	9	

Statistical analyses were performed by the χ2 test.

TNM, tumor‐node‐metastasis.

^*^ *P* < 0.05 was considered significant.

### Cell culture

Human normal lung epithelial cells BEAS‐2B and LAD cell line A549 were purchased from American Tissue Culture Collection (ATCC, Manassas, VA, USA). These cells were seeded in RPMI‐1640 medium A1049101, Gibco, Grand Island, NY, USA) with 10% FBS and incubated in a humid atmosphere with 5% CO_2_ at 37°C.

### Cell transfection

Short hairpin RNA targeting ZFAS1 (5′‐CTG GCT GAA CCA GTT CCA CAA GGT T‐3′) and FRS2 (5′‐TAC TTC TCC TAG TTG CAG TCA GG‐3′), ZFAS1 overexpression vector and miR‐1271‐5p mimics (5′‐CUU GGC ACC UAG CAA GCA CUC A‐3′) or inhibitor (5′‐UGA GUG CUU GCU AGG UGC CAA G‐3′) were purchased from GeneChem (Shanghai, China). Lipofectamine 2000 (Invitrogen/Thermo Fisher Scientifc) was used to transfect them into A549 cells.

### RT‐qPCR

The TRIzol Reagent (15 596 018, Thermo Fisher Scientific, Waltham, MA, USA) was used to acquire total RNA in LAD tissues and cells. The synthesis of the first‐strand complementary DNA was synthesized by the MiRNA Reverse Transcription kit (4 366 597, Thermo Fisher Scientific). RT‐qPCR was performed using SYBR Premix Dimer Eraser Kit (RR091A, Takara, Dalian, China). The expressions of ZFAS1, miR‐1271‐5p and FRS2 were analyzed by 2^‐ΔΔCt^ method, and GAPDH or U6 snRNA served as an internal control. The primers used were: ZFAS1 forward 5′‐ACG TGC AGA CAT CTA CAA CCT‐3'and reverse 5′‐TAT TCC AAC ACC CGC AT‐3′; miR‐1271‐5p forward: 5′‐CTT GGC ACC TAG CAA GCA CTC A‐3′ and reverse, 5′‐CCA GTG CAG GGT CCG AGG T‐3′; U6 forward: 5′‐GCT TCG GCA GCA CAT ATA CTA AAA T‐3′ and reverse, 5′‐CGC TTC ACG AAT TTG CGT GTC AT‐3′; FRS2 forward: 5′‐GTG CCG CAT CTT TAC CCT CA‐3′ and reverse, 5′‐TCG CCA TTA AAT TCT GGC TGC‐3′; GAPDH forward: 5′‐ACA ACT TTG GTA TCG TGG AAG G‐3′, and reverse, 5′‐GCC ATC ACG CCA CAG TTT C‐3′.

### Western blot analysis

Protein samples were lysed using RIPA buffer (Beyotime, Shanghai, China). Then, we collected the supernatant as the total proteins. The proteins were electrophoresed by 10% SDS‐PAGE and then blocked with 5% nonfat milk for one hour. After incubating the protein with the following primary antibodies (FRS2 and GAPDH) overnight at 4°C, the diluted secondary antibodies was added to incubate protein for another one hour. Finally, the protein was examined using ECL reagent (Millipore, MA, USA).

### CCK‐8 assay

Transfected A549 cells (2 × 10^3^ cells/well) were seeds in 96‐well plates and incubated for 24, 48, 72 or 96 hours in RPMI‐1640 medium, respectively. Next, all the cells were incubated with 10 μL CCK‐8 reagents for four hours. The medium was discarded and dimethyl sulfoxide was added. After 10 minutes shaking, a Microplate Absorbance Reader (Thermo Fisher Scientific) was used to evaluate the color reaction at 450 nm.

### Transwell assay

Tumor cell migration and invasion assay was performed in a 24‐well transwell chamber (8 um pore size polycarbonate membrane filter, Corning, New York), which was coated with or without Matrigel (Becton‐Dickinson, Bedford, Massachusetts). After 48 hours, a single‐cell suspension was prepared with trypsinization and the density was adjusted to 2 × 10^4^ cells/mL. Then, 200 μL of the cell suspension were seeded in the upper chambers and incubated in 500 μL RPMI‐1640 medium without FBS, while 500 μL medium with 10% FBS was placed in the lower chambers. The plates were incubated for 24 hours in a 5% CO_2_ humidified incubator at 37°C. Cells on the upper side of the filters were removed with cotton‐tipped swabs. Next, the cells on the lower side were fixed in 4% formaldehyde and stained with 1% crystal violet in PBS for five minutes at room temperature. The cells on the lower side of the filters were defined as migration or invasion cells and counted at × 200 magnification in five random fields of each filter.

### Dual luciferase reporter assay

The pGL3 plasmid with wt‐ZFAS1 or mut‐ZFAS1 and pGL3 plasmid with wt‐FRS2 or mut‐FRS2 were cotransfected into A549 cells containing miR‐1271‐5p mimics. Renilla luciferase reporter pRL‐CMV (Promega, Madison, Wisconsin) was transfected into cells as a normalizing transfection control. After 48 hours, the activities of firefly and renilla luciferases were measured using the Dual Luciferase Assay Kit (Promega) with reference to the protocols of manufacturer. Normalized data were calculated as the quotient of renilla/firefly luciferase activities.

### Statistical analysis

All data were obtained from at least three independent experiments with a similar pattern. GraphPad Prism 6.0 and SPSS 17.0 were used to perform statistical analysis. The χ2 test was performed to analyze the association between the expression of ZFAS1 and patient clinical data. Pearson's analysis was used for correlation analysis. Differences between groups were calculated using Student's *t*‐test or one‐way ANOVA followed by Tukey's post hoc test. Data are shown as mean ± SD. Differences with *P* < 0.05 were considered statistically significant.

## Results

### Knockdown of ZFAS1 suppresses cell proliferation, migration and invasion in LAD

**Figure 1:**
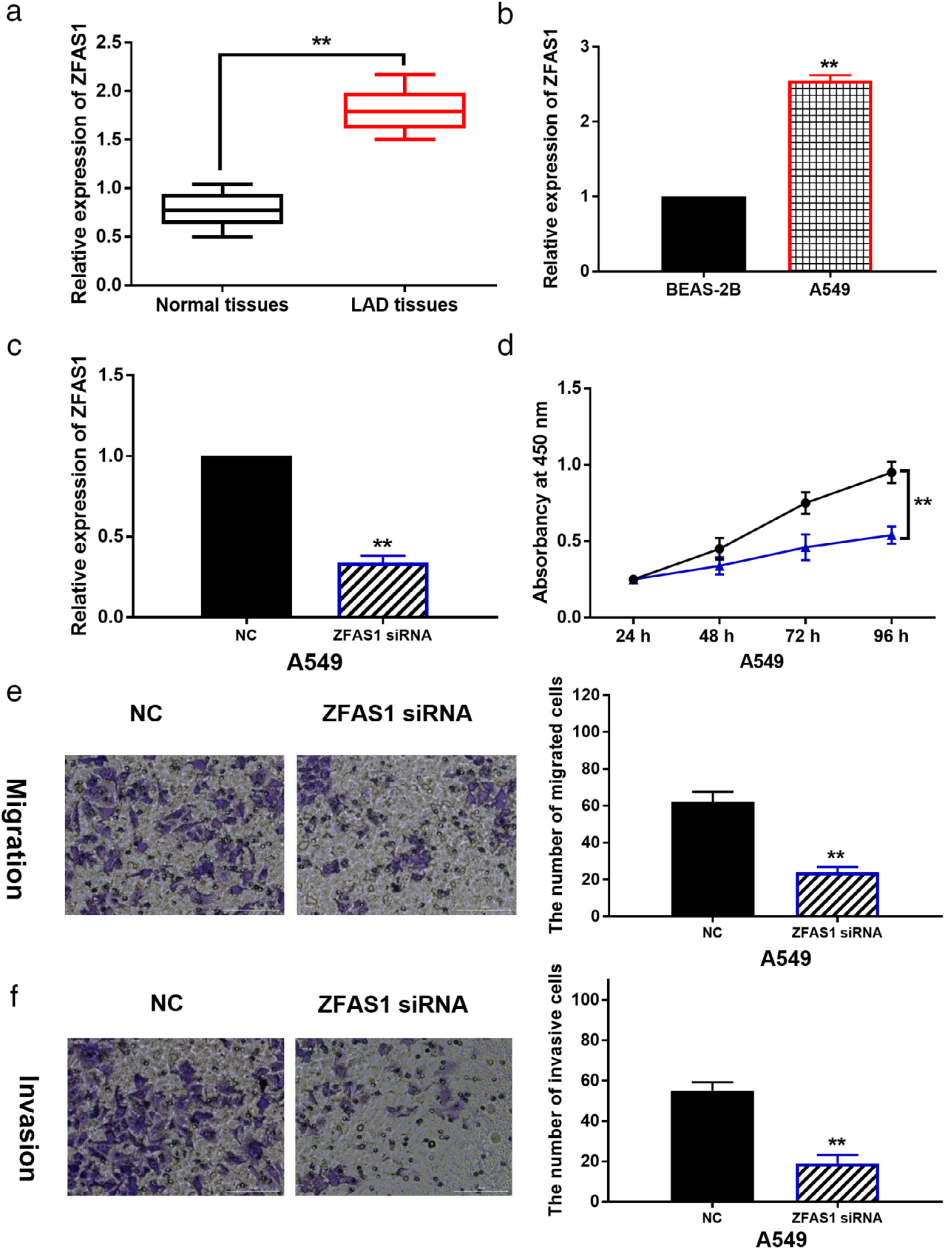
LncRNA ZFAS1 aggravates the malignancy of LAD. (**a**) The expression of ZFAS1 in LAD tissues and normal tissues. (**b**) ZFAS1 expression in A549 and BEAS‐2B cells. (**c**) The expression of ZFAS1 in A549 cells with its siRNA. (**d**, **e**, **f**) Cell proliferation, migration and invasion in A549 cells with ZFAS1 siRNA **P < 0.01 (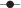) NC, (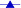)" ZFAS1 siRNA.

First, the abnormal expression of lncRNA ZFAS1 was detected in LAD by RT‐qPCR. We found that lncRNA ZFAS1 was upregulated in LAD tissues compared to normal tissues (Fig 1a). In addition, abnormal expression of lncRNA ZFAS1 was related to TNM stage and lymph node metastasis in LAD patients (Table 1). Meanwhile, lncRNA ZFAS1 expression in A549 cells was higher than that in BEAS‐2B cells (Fig 1b). To explore the role of lncRNA ZFAS1 in LAD, ZFAS1 siRNA was transfected into A549 cells. RT‐qPCR showed that ZFAS1 siRNA significantly reduced ZFAS1 expression in A549 cells (Fig 1c). CCK‐8 showed that cell proliferation was inhibited by ZFAS1 downregulation in A549 cells (Fig 1d). Transwell assay suggested that knockdown of ZFAS1 suppressed cell migration and invasion in A549 cells (Fig 1e, f). These results indicate that upregulation of lncRNA ZFAS1 aggravates the malignancy of LAD.

### LncRNA ZFAS1 acts as a molecular sponge of miR‐1271‐5p

**Figure 2:**
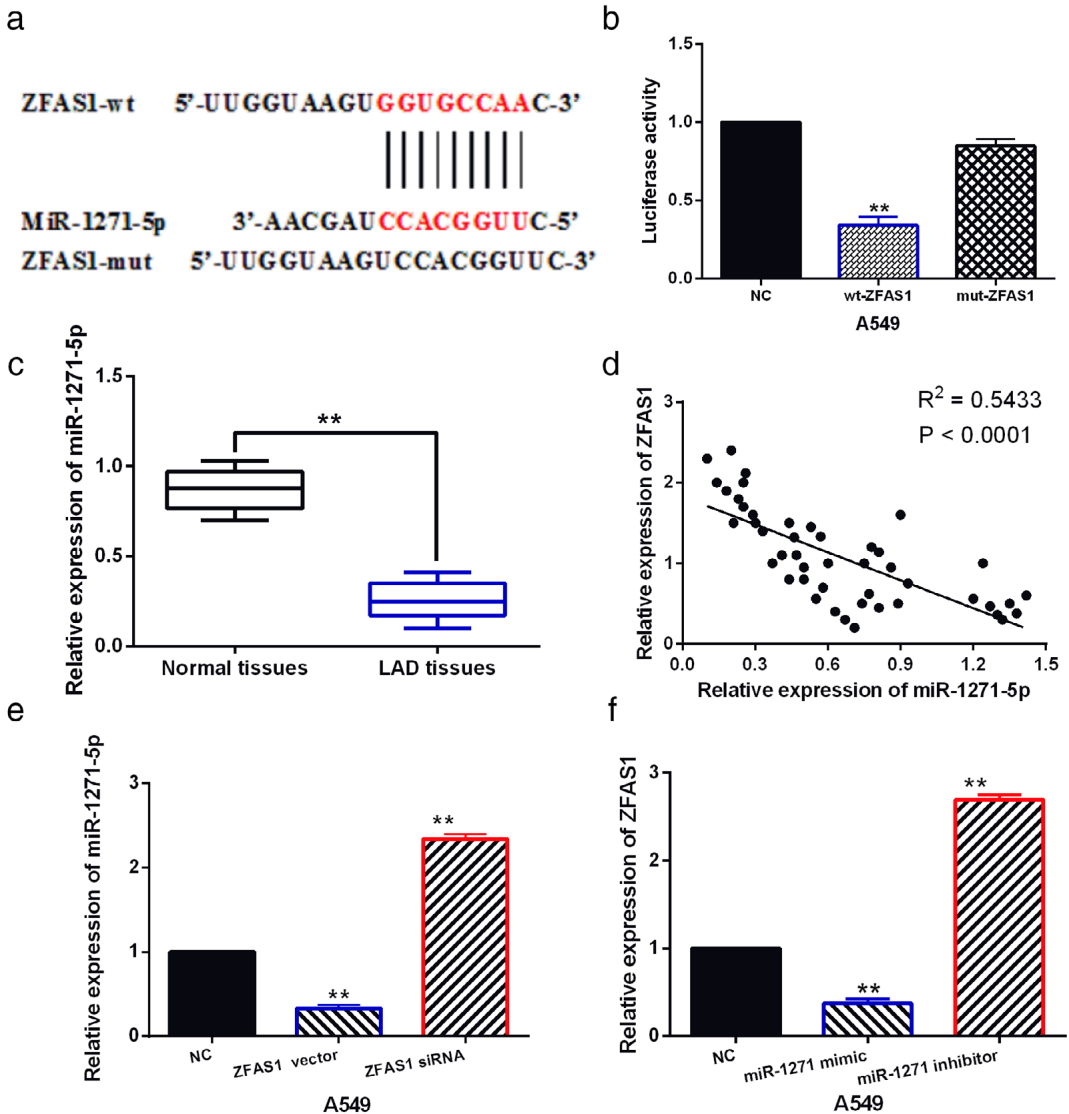
LncRNA ZFAS1 acts as a molecular sponge of miR‐1271‐5p. (**a**) The binding sites between ZFAS1 with miR‐1271‐5p. (**b**) Luciferase reporter assay. (**c**) MiR‐1271‐5p expression in LAD tissues and normal tissues. (**d**) A negative correlation was found between ZFAS1 and miR‐1271‐5p expression in LAD tissues. (**e**) MiR‐1271‐5p expression in A549 cell with ZFAS1 siRNA and vector (**f**) ZFAS1 expression in A549 cells containing miR‐1271‐5p mimics or inhibitor **P < 0.01.

The starBase version 2.0 (http://starbase.sysu.edu.cn/) predicts that lncRNA ZFAS1 has a binding site with miR‐1271‐5p (Fig 2a). To verify this prediction, dual luciferase reporter assay was performed in A549 cells. We found that miR‐1271‐5p mimics reduced the luciferase activity of wt‐ZFAS1 in A549 cells (Fig 2b), indicating that miR‐1271‐5p could directly bind to lncRNA ZFAS1. To explore whether miR‐1271‐5p is involved in LAD progression, miR‐1271‐5p expression was detected in LAD. MiR‐1271‐5p expression was found to be decreased in LAD tissues compared to normal tissues (Fig 2c), and a negative correlation between lncRNA ZFAS1 and miR‐1271‐5p expression was detected in LAD tissues (Fig 2d). In A549 cells, miR‐1271‐5p expression was decreased by ZFAS1 vector and increased by ZFAS1 siRNA (Fig. 2e). Meanwhile, miR‐1271‐5p overexpression inhibited ZFAS1 expression, while miR‐1271‐5p downregulation promoted ZFAS1 expression in A549 cells (Fig 2f). Taken together, lncRNA ZFAS1 can directly bind to miR‐1271‐5p, and lncRNA ZFAS1 acts as a molecular sponge of miR‐1271‐5p in LAD.

### MiR‐1271‐5p inhibits cell proliferation, migration and invasion in LAD by regulating lncRNA ZFAS1

**Figure 3:**
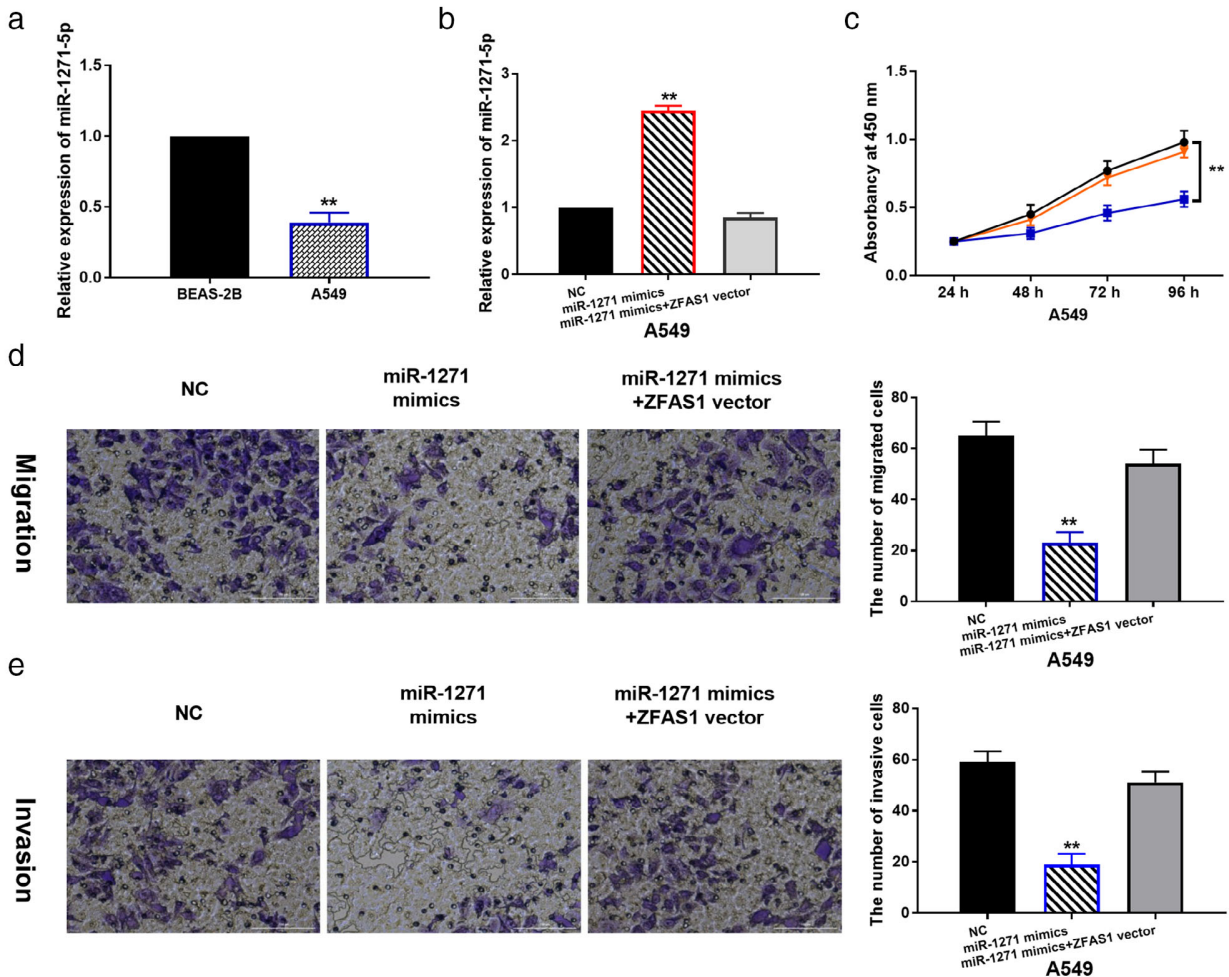
MiR‐1271‐5p inhibits LAD progression by regulating lncRNA ZFAS1. (**a**) The expression of miR‐1271‐5p in A549 and BEAS‐2B cells. (**b**) MiR‐1271‐5p expression in A549 cells with miR‐1271 mimics, or miR‐1271 mimics + ZFAS1 siRNA. (**c**, **d**, **e**) Cell proliferation, migration and invasion in A549 cells with miR‐1271 mimics, or miR‐1271 mimics+ ZFAS1 siRNA ** *P* < 0.01 (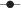) NC, (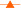) miR‐1271 mimics + ZFAS1 vector, (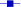) miR‐1271 mimics.

Next, downregulation of miR‐1271‐5p was found in A549 cells contrast to BEAS‐2B cells (Fig 3a). Then, ZFAS1 vector was transfected into A549 cells containing miR‐1271‐5p mimics to explore their relationship in LAD. RT‐qPCR suggested that miR‐1271‐5p mimics enhanced its expression in A549 cells. However, ZFAS1 vector reduced the increased expression of miR‐1271‐5p in A549 cells (Fig 3b). Meanwhile, we found that miR‐1271‐5p overexpression suppressed cell proliferation, migration and invasion in A549 cells, and upregulation of ZFAS1 also abolished the inhibitory effect of miR‐1271‐5p on cell proliferation, migration and invasion in A549 cells (Fig 3c–e). The results imply that miR‐1271‐5p functions as a tumor suppressor in LAD, and lncRNA ZFAS1 promotes LAD development by downregulating miR‐1271‐5p.

### FRS2 is a direct target of miR‐1271‐5p

**Figure 4:**
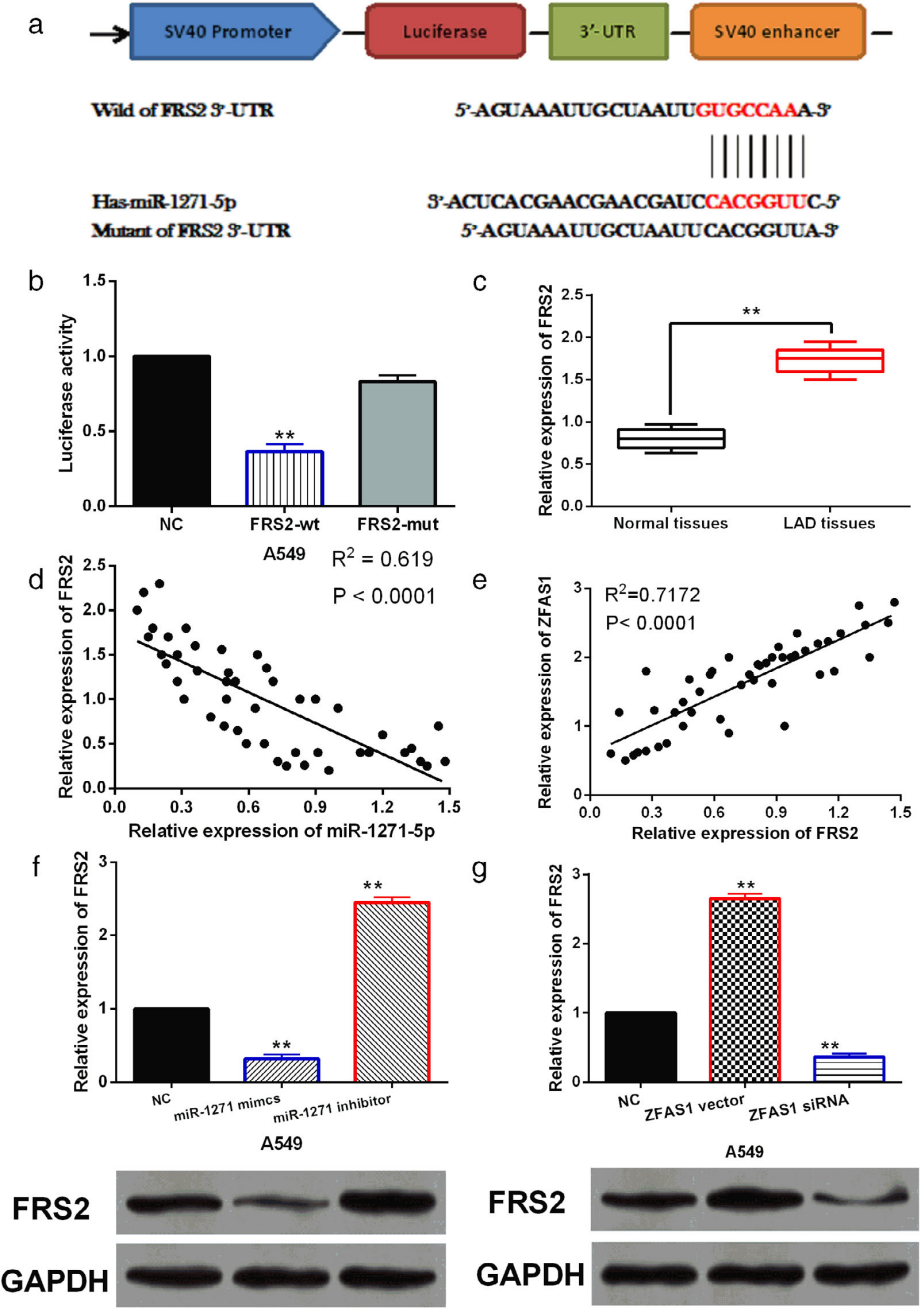
FRS2 is a direct target of miR‐1271‐5p. (**a**) The binding sites between miR‐1271‐5p and FRS2. (**b**) Luciferase reporter assay. (**c**) FRS2 expression in LAD tissues and normal tissues. (**d**) A negative correlation was found between FRS2 and miR‐1271‐5p expression in LAD tissues. (**e**) A positive correlation was found between ZFAS1 and FRS2 expression in LAD tissues. (**f**) The mRNA and protein expression of FRS2 in A549 cells with miR‐1271‐5p mimics or inhibitor. (**g**) The mRNA and protein expression of FRS2 in A549 cells with ZFAS1 siRNA or vector ** *P* < 0.01.

Further, the downstream target of miR‐1271‐5p was searched in TargetScan database (http://www.targetscan.org). It predicts that FRS2 may be a potential target of miR‐1271‐5p (Fig 4a). Dual luciferase reporter assay showed that miR‐1271‐5p mimics reduced the luciferase activity of wt‐FRS2 and had little effect on mut‐FRS2 luciferase activity in A549 cells (Fig 4b), indicating that miR‐1271‐5p directly targets FRS2. Next, the abnormal expression of FRS2 was detected in LAD. We found that FRS2 expression was upregulated in LAD tissues (Fig 4c), and FRS2 was inversely correlated with miR‐1271‐5p expression in LAD tissues (Fig 4d). But lncRNA ZFAS1 was positively associated with FRS2 expression in LAD tissues (Fig 4e). Consistently, miR‐1271‐5p mimics reduced FRS2 expression, while miR‐1271‐5p inhibitor upregulated FRS2 expression in A549 cells (Fig 4f). Meanwhile, the mRNA and protein expression of FRS2 was enhanced by ZFAS1 upregulation and declined by ZFAS1 downregulation in A549 cells (Fig 4g). The above results reveal that FRS2 is a direct target of miR‐1271‐5p, and lncRNA ZFAS1 can positively regulate FLOT2 expression in LAD.

### FRS2 promotes cell proliferation, migration and invasion in LAD by participating in lncRNA ZFAS1/miR‐1271‐5p axis

**Figure 5:**
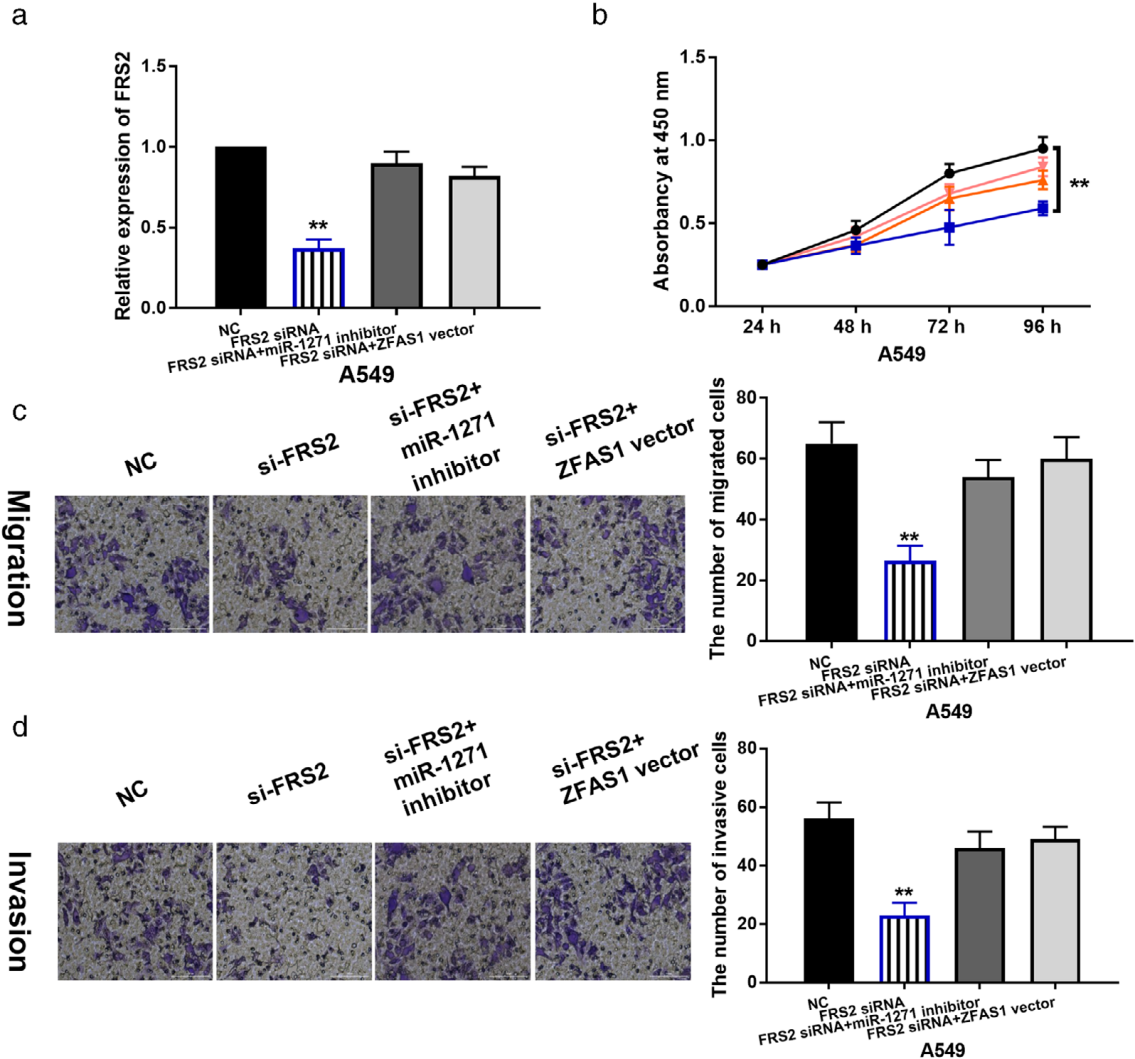
FRS2 regulates the development of LAD by participating in lncRNA ZFAS1/miR‐1271‐5p axis. (**a**) FRS2 expression in A549 cells with FRS2 siRNA, FRS2 siRNA + miR‐1271 inhibitor or FRS2 siRNA + ZFAS1 vector. (**b**, **c**, **d**) Cell proliferation, migration and invasion in A549 cells with FRS2 siRNA, FRS2 siRNA + miR‐1271 inhibitor or FRS2 siRNA + ZFAS1 vector ** *P* < 0.01 (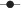) NC, (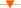) FRS2 siRNA + miR‐1271 inhibitor, (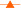) FRS2 siRNA + ZFAS1 vector, (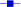) FRS2 siRNA.

To confirm whether FRS2 is involved in LAD progression by interacting with ZFAS1 and miR‐1271‐5p, ZFAS1 vector or miR‐1271 inhibitor was transfected into A549 cells with FRS2 siRNA. FRS2 expression was found to be reduced by its siRNA in A549 cells. However, the downregulation of FRS2 induced by its siRNA was restored by ZFAS1 vector or miR‐1271‐5p inhibitor in A549 cells (Fig 5a). Functionally, cell proliferation, migration and invasion were found to be restrained by knockdown of FRS2 in A549 cells, and lncRNA ZFAS1 upregulation or miR‐1271‐5p downregulation weakened the inhibitory effect of FRS2 siRNA on cell proliferation, migration and invasion in A549 cells (Fig 5b–d). These results imply that FRS2 promoted progression of LAD by mediating lncRNA ZFAS1/miR‐1271‐5p axis.

## Discussion

Today, the role of lncRNAs in LAD is being increasingly recognized. For example, LINC01419 was found to be upregulated in LAD, and promoted LAD cell proliferation and metastasis.^17^ In this study, upregulation of lncRNA ZFAS1 was detected in LAD tissues and cells. Functionally, knockdown of lncRNA ZFAS1 restrained cell proliferation, migration and invasion in LAD cells. Consistently, upregulation of lncRNA ZFAS1 has been found in acute promyelocytic leukemia and hepatocellular carcinoma.^18^, ^19^ Wang *et al*. reported that lncRNA ZFAS1 promoted cell proliferation and migration and inhibited apoptosis in nasopharyngeal carcinoma in vitro,^20^ and upregulation of lncRNA ZFAS1 promoted cell migration and invasion in clear cell renal cell carcinoma.^21^ More importantly, knockdown of lncRNA ZFAS1 was found to suppress NSCLC progression via targeting miR‐150‐5p/HMGA2 signaling.^22^ These results are consistent with our study, indicating that lncRNA ZFAS1 serves as an oncogene in LAD. However, the effect of lncRNA ZFAS1 on in vivo tumor growth has not been investigated in this study. Thus, further validation still needs to be done in the future.

Previous studies have shown that lncRNA ZFAS1 regulate tumorigenesis of human cancers by interacting with miRNAs, such as miR‐193a‐3p and miR‐590‐3p.^23^, ^24^ In our research, we found that lncRNA ZFAS1 directly binds to miR‐1271‐5p. Overexpression of miR‐1271‐5p inhibited cell proliferation, migration and invasion in LAD. It has been shown that miR‐1271‐5p was decreased in ovarian cancer and breast cancer.^25^, ^26^ The inhibitory effect of miR‐1271 on cell proliferation and invasion has also been identified in colorectal cancer.^27^ LncRNA MALAT1 facilitated the tumorigenesis, invasion and glycolysis of multiple myeloma via miR‐1271‐5p/SOX13 axis.^28^ Here, lncRNA ZFAS1 was also found to promote LAD development by downregulating miR‐1271‐5p. All these results suggest that miR‐1271‐5p acts as a tumor inhibitor in LAD, and miR‐1271‐5p regulates LAD development by interacting with lncRNA ZFAS1. However, this study only preliminarily explains the regulatory mechanism of lncRNA ZFAS1/miR‐1271‐5p in LAD. The specific interaction between lncRNA ZFAS1 and miR‐1271‐5p still needs to be explored in the future.

Further, FRS2 was confirmed to be a direct target of miR‐1271‐5p. Upregulation and carcinogenesis of FRS2 was observed in LAD. Similar to our results, high expression and carcinogenesis of FRS2 was found in Wilms' tumor and gastric cancer.^29^, ^30^ MiR‐613 attenuated the proliferation, migration and invasion of Wilms' tumor cells via targeting FRS2.^31^ Consistently, miR‐1271‐5p restrained the development of LAD by inhibiting FRS2 expression. In addition, it has been demonstrated that lncRNA ANRIL promoted cell proliferation and migration via sponging miR‐339‐5p and regulating FRS2 expression in atherosclerosis.^32^ This study showed that lncRNA ZFAS1 may promote LAD progression by sponging miR‐1271‐5p and upregulating FRS2.

In conclusion, upregulation of lncRNA ZFAS1 was identified in LAD tissues and cells. Functionally, lncRNA ZFAS1 promotes cell proliferation, migration and invasion in LAD by downregulating miR‐1271‐5p or upregulating FRS2. However, the results are only verified in vitro. Therefore, further in vivo studies are still essential.

## Disclosure

The authors declare that they have no competing interests.
